# Dynamic Evaluation of Patellofemoral Instability: A Clinical Reality or Just a Research Field? A Literature review

**DOI:** 10.1111/os.12549

**Published:** 2019-12-02

**Authors:** Sergio Barroso Rosa, Peter Mc Ewen, Kenji Doma, Juan Francisco Loro Ferrer, Andrea Grant

**Affiliations:** ^1^ The ORIQL, Orthopaedic Research Institute of Queensland Townsville (QLD) Australia; ^2^ Clinical Sciences Department University of Las Palmas de Gran Canaria Las Palmas de Gran Canaria Canary Islands Spain; ^3^ College of Healthcare Sciences James Cook University Townsville (QLD) Australia

**Keywords:** Patellofemoral joint, Patellar dislocation, Muscle contraction, Movement, Diagnostic techniques, Procedures

## Abstract

Patellofemoral instability (PFI) is one of the most disabling conditions in the knee, often affecting young individuals. Despite its not uncommon presentation, the underlying biomechanical features leading to this entity are not entirely understood. The suitability of classic physical examination manoeuvres and imaging tests is a matter of discussion among treating surgeons, and so are the findings provided by these means. A potential cause for this lack of consensus is the fact that, classically, the diagnostic approach for PFI has relied on statically obtained data. Many authors advocate for the study of this entity in a dynamic scenario, closer to the actual situation in which the instability episodes occur. In this literature review, we have compiled the available data from the last decades regarding dynamic evaluation methods for PFI and related conditions. Several categories are presented, grouping the related techniques and devices: physical examination, imaging modalities (ultrasound (US), magnetic resonance imaging (MRI), computed tomography (CT) and combined methods), arthroscopic evaluation, and others. In conclusion, although a vast number of quality studies are presented, in which comprehensive data about the biomechanics of the patellofemoral joint (PFJ) are described, this evidence has not yet reached clinical practice universally. Most of the data still stays in the research field and is seldom employed to assist a better understanding of the PFI cases and their ideal treatment targets.

## Introduction

The patellofemoral joint (PFJ), despite its apparent minor contribution to knee biomechanics, remains to be one of the less understood components in the lower limb. As several musculoskeletal conditions associated with the PFJ (e.g. arthritis, anterior knee pain syndrome) are troublesome in terms of diagnosis and successful treatment, there is a growing interest on patellofemoral instability (PFI)^1^.

PFI is defined as an abnormal patellar tracking in relation to the femoral trochlea as the knee extends/flexes. Subtle cases present with discomfort during prolonged knee flexion or at certain sporting activities. In more severe situations, PFI can lead to recurrent patellar dislocation, a disabling condition. The list of predisposing factors recognized for PFI is vast, including (but not limited to): patella alta, trochlear dysplasia, increased Q‐angle, muscular imbalance, increased femoral‐tibial torsion, genu valgus, hyperlaxity, and traumatic rupture of stabilizers such as the medial patellofemoral ligament^2^, ^3^, ^4^, ^5^.

Proper diagnosis of PFI requires a thorough physical examination with comprehensive imaging studies. However, there is still no consensus on appropriate techniques and measurements for PFI diagnosis based on previous literature. As a result, surgeons are often left with selecting diagnostic procedures according to personal preference, rather than utilizing evidence‐based practice^6^. Furthermore, and perhaps more concerning, is that surgical correction is generally planned according to the data obtained from these equivocal investigations.

**Figure 1:**
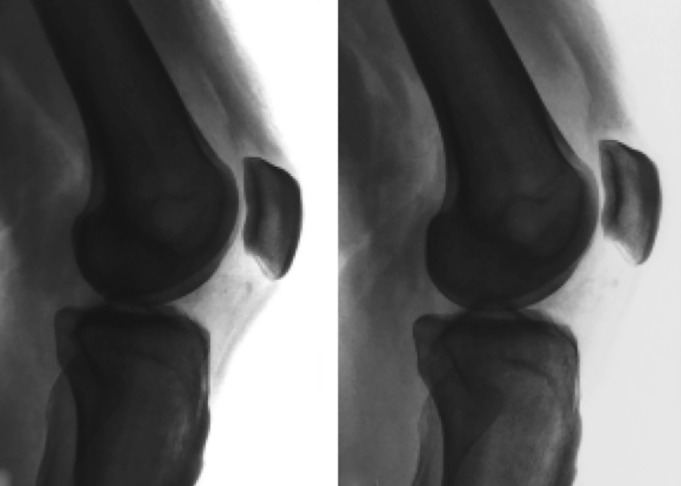
Effect of weight bearing (WB) (image on the right) on patellar proximal displacement in lateral radiographs^7^.

Patellar dislocation episodes typically occur during movement, in early degrees of flexion, when constraint provided by the femoral trochlea is less effective. Only in extreme cases can the patella dislocate while the patient is resting in a sitting or recumbent position, but fortunately this is not a common presentation. However, most currently applied physical examination manoeuvres and imaging tests are performed in this scenario: a supine patient in a resting attitude, rather than exploring the knee in more instability‐prone conditions. Does this mean we may be building the whole diagnostic and therapeutic process from an incorrect starting point? Singularly, the widely employed classic patellar height ratios (Insall‐Salvati, modified Insall‐Salvati, Blackburne‐Peel, Caton‐Deschamps) change significantly if obtained during weight bearing (WB)^7^ (Fig. 1).

Many authors advocate for the study of the PFJ from a dynamic point of view. Presumably, reproducing the situations in which instability occurs should provide clinicians with a more relevant assessment of patellar mechanics. This trend is not recent, and several related diagnostic papers have been published in the last decades, as will be presented in this review. Moreover, additional papers have pointed out the utility of dynamic techniques in the postoperative evaluation of PFI surgical correction results. The objective of this review is to summarize the available evidence of the dynamic assessment of PFI; can it be considered a clinical tool, or does it remain a field in need of further research?

Muhle *et al*. stated that ideal dynamic tests should be taken under WB conditions, while actively performing functional tasks such as walking, stepping, or squatting, in a short time, and with assumable costs (Table 1)^8^. Under the banner of *dynamic* evaluation, we have encountered a variety of concepts. Some reports refer to tests performed with active participation of the subject under WB, while others evaluate the PFJ under isometric quadriceps contraction. To avoid confusion, the term *kinematic* is often employed in publications involving active movement.

**Table 1:** Muhle *et al*. criteria for an ideal test for dynamic assessment of the patellofemoral joint (PFJ)^8^

Visualization of full range of patellar motion.
Active movement
Weight bearing (WB) conditions
Avoidance of radiation or invasive techniques
Short examination time
Reproducible
Clear view of the PF joint and the femoral condyles, specially in the axial view
Good image quality
Low cost
High availability
3D imaging for patellar tracking

A first observation coming from the revised literature would be the fact that during the past decades, large amounts of research have been conducted in order to study the PFJ joint under dynamic conditions (Table 2). Nevertheless, despite several key findings, most research lines seem to run independently, without integrating previous data. This could be explained by the heterogeneity among publications: diversity of imaging devices, processing protocols, patient positioning, active or passive motion (among other factors). The amount of information is considerable but may be hard to integrate.

**Table 2:** Summary of available literature in PFJ dynamic evaluation

Author	Year	Category	Participants	Findings and comments
Beckert *et al*.^9^	2016	PE	10 PFI patients	MRI more accurate than clinical J‐sign for patellar position
				Suggests lateral patella edge as a better target than static TTTG for surgical correction
Sarkar *et al*.^29^	2009	PE	23 healthy women	Relevant changes in Q angle with quads contraction
Shih *et al*.^27^	2004	US	10 healthy controls	Significant changes in patellar tilt between sitting, squatting and stepping
Shellock *et al*.^40^	1988	MRI	1 PFI patient	Sequential static MRI slices at different degrees of flexion to produce a kinematic sequence
			Four healthy controls	
Brossman *et al*.^30^	1993	MRI	13 maltracking cases	First motion‐triggered report
			15 healthy controls	
Sheehan *et al*.^23^	1999	MRI	18 healthy knees	First published cine‐phase study
Witonski and Góraj^10^	1999	MRI	12 AKP cases	Different values for most parameters in the PFJ if obtained under relaxed conditions *vs*. quadriceps activation
			20 healthy controls	
McNally *et al*.^41^	2000	MRI	474 AKP cases	First ultrafast MRI article
Draper *et al*.^24^	2009	MRI	13 AKP women	Assessment of bracing effect on patellar tilt and subluxation
			14 healthy women	
Carlson *et al*.^44^	2017	MRI	32 AKP cases	Static TTTG does not correlate with lateral tracking at full extension
			38 healthy controls	
Burke *et al*.^45^	2018	MRI	20 PFI cases	First use of real time gradient echo imaging in peripheral skeleton
			10 healthy controls	Suggests patellar subluxation greater than 3 mm as highly specific for PFI
Barroso *et al*.^26^	2018	MRI	9 PFI cases	Dynamic assessment method of patellar height
			68 controls	
Dupuy *et al*.^36^	1997	CT	20 AKP knees	First report on spiral CT
				Higher sensitivity than static sequences
Elias *et al*.^37^	2014	CT	6 PFI	Dynamic assessment after surgical stabilization
Tanaka *et al*.^39^	2016	CT	67 PFI knees	Maltracking grading system
Nha *et al*.^28^	2008	Misc	Eight healthy controls	Validated method combining static MRI + 2D fluoroscopy during weight bearing (WB)
				List of normal values for several PFJ features
Liu *et al*.^56^	2017	Misc	30 PFI cases	Diffusor tensor imaging as an early detector of potential PFI cases
			30 controls	
Wilson *et al*.^54^	2009	Misc	10 PTI patients	Thermoplastic patellar clamp and optoelectronic motion capture
			10 healthy controls	
Suganuma *et al*.^16^	2014	Misc	24 PFI knees	Arthroscopically observed that quads activation significantly alters the relations in the PFJ
			49 controls	

CT, computed tomography; PE, physical examination; PFI, patellofemoral instability; Misc, miscellaneous; MRI, magnetic resonance imaging; US, ultrasound.

**Figure 2:**
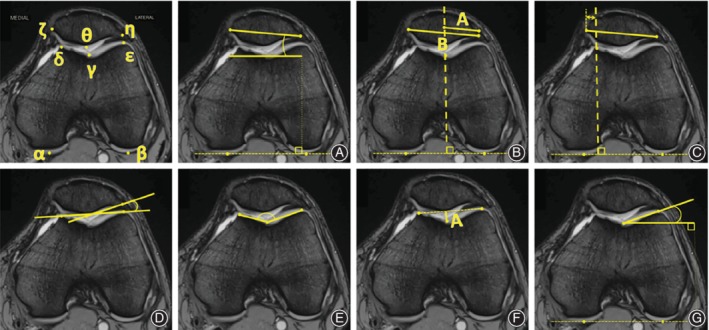
Patellofemoral measurements and angles most commonly obtained. Reference points on upper left diagram: Most posterior aspect of medial (α) and lateral (β) femoral condyles, deepest trochlear point (γ), most anterior aspect of medial (δ) and lateral (ε) femoral condyles, medial (ζ) and lateral (η) patellar borders, intersection of medial and lateral patellar facets (θ). (A) Patellar tilt: angle between αβ and ζη. (B) Bisect offset: A/B x 100 (dashed line intersecting ϒ). (C) Lateral patellar displacement: positive value towards lateral side, negative to medial (dashed line intersecting δ). (D) Lateral patellofemoral angle: angle between δε and θη. (E) Sulcus angle: between δγ and γε. (F) Sulcus depth: distance from γ to δε. (G) Lateral trochlear inclination: angle between αβ and γε.

Another key point in this review is that, significantly, several authors question the validity of data obtained from static examinations. Routine findings obtained in a first contact with the patient such as an increased *static* Q angle or the presence of a positive j‐sign may not be that relevant^9^, ^29^. Still, conventional imaging measures, such as patellar tilt or sulcus angle, vary whether the images are obtained statically or not^10^ (Fig. 2). Some studies even observed that passive motion of the joint was not sufficient to reproduce the abnormal biomechanics of unstable joints^30^, and claim for the risk of obtaining false negative results with static magnetic resonance imaging (MRI) protocols^31^.

An additional interesting observation is that patellar tracking is a recurring item of discussion among the quoted references. The definition of a normal patellar tracking is inherently confusing, as the movement itself is complex, and the references to define it vary depending on the authors. Likewise, patellar tracking is often not assessed during WB, ignoring a crucial element in the physiological knee dynamics.

A common feature of the reported articles' results is that most evidence comes from small cohorts, in certain cases implying just healthy volunteers. This limits the diagnostic utility of the results and could explain why very few publications report specific figures with practical use as cut‐off points in clinical decision making.

## Methods

A literature review was conducted by the first author (SB). Search terms included *patellar instability, patellar dislocation, dynamic, kinematic, active, assessment, diagnostic*, and *imaging*. PubMed was the primary search source, and side searches were conducted in private databases from associated universities and scientific organizations. No limit for publication date was set.

Publications were considered if they reported any sort of assessment method for patellar biomechanics in the setting of patellar instability with no‐static conditions. Cadaveric, animal, or virtual/computerized models were also rejected. Papers in languages other than English or Spanish were also discarded.


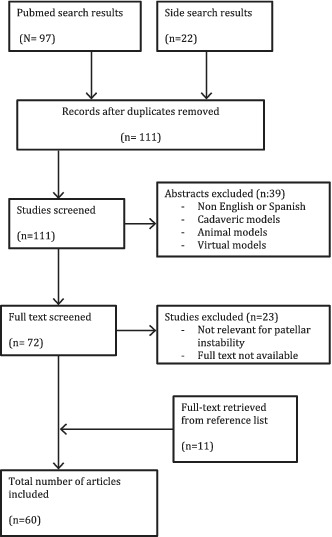


## Review Results

### *Physical Examination*

**Figure 3:**
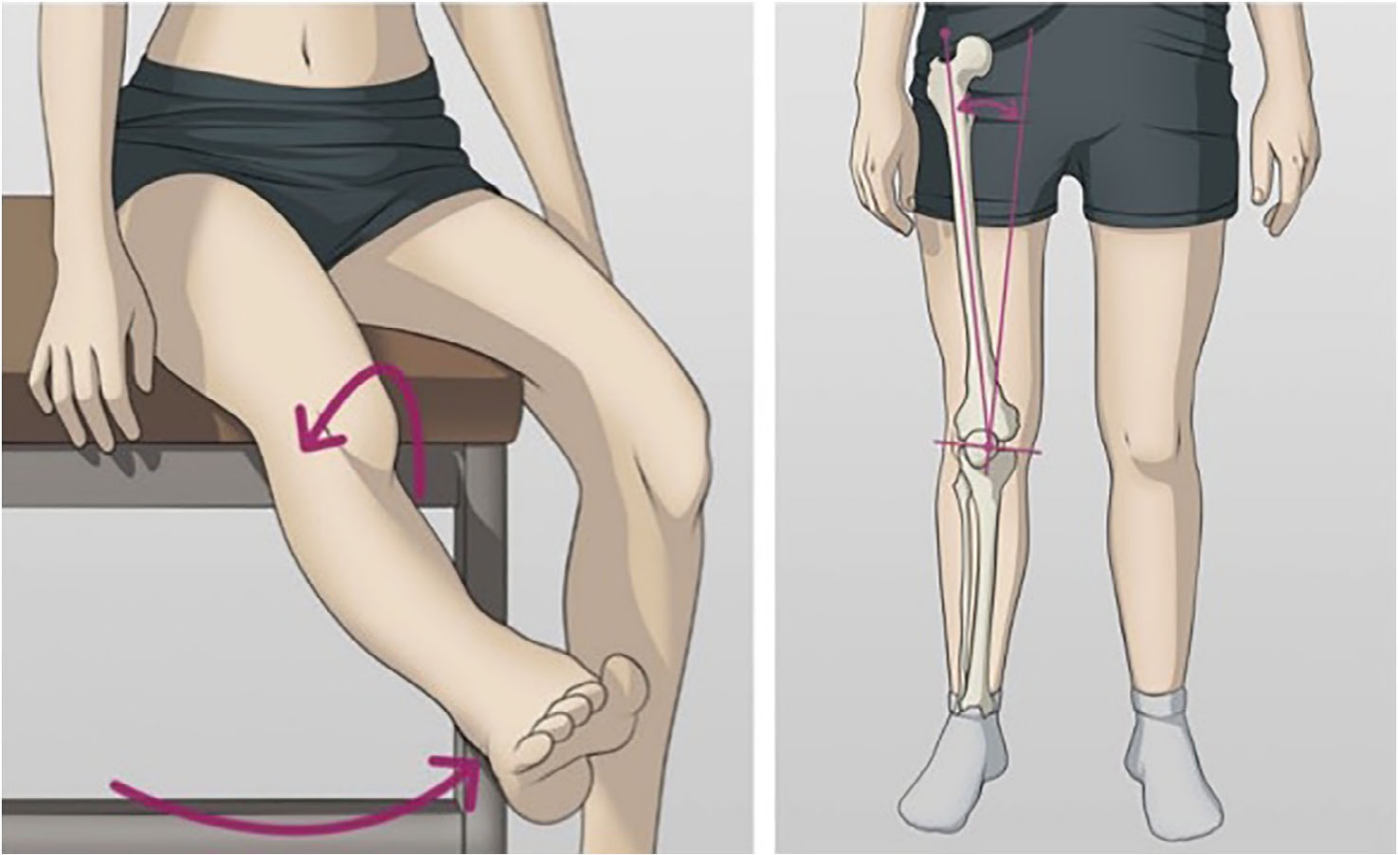
J‐sign and Q‐angle. Reproduced with authorization of Medisavvy.

A classical examination of a PFI patient takes place with the subject laying or sitting on the examination table, while the knee is passively explored. As an illustrative example of this we can cite “*Comprehensive Physical Examination for Instability of the Knee*”^11^, an instructive manuscript with 18 pages of manoeuvres where an only dynamic test is presented for PFI: evaluation of the *j‐sign* (Fig. 3).

The diagnostic utility of the *j‐sign* has even been questioned. In a cohort of PFI patients, Beckert *et al*. found poor correlation between *j‐sign* and a centered position of the patella in relation to the trochlear groove^9^. Another review on patellar tracking identified multiple limitations in defining specific movement patterns and questions its diagnostic suitability^32^.

Sarkar *et al*.^29^ investigated the influence of quadriceps muscle activation on the Q angle, another classic examination item in PFJ assessment; observing a statistically significant difference of 4.65° ± 2.74° between relaxed and quads contracted (IQA) measurements. Therefore, they concluded that: “*Measuring the change in the Q angle with IQA compared with the resting Q angle may enhance a clinicianʼs ability to predict which individual is at greater risk of developing patellar tracking and patellar dysfunction*.” Türkmen *et al*. calculated the average Q angle between different positions (standing, sitting, and supine) with and without quadriceps contraction, obtaining a ΔQ angle which was significantly lower in PF pain cases^12^.

A recently published paper^13^ described the novel “*reverse dynamic patellar apprehension test*,” in which the PFJ stability is explored under a medially applied force from deep flexion to extension, as opposed to the classic apprehension test where the exploration begins in full extension. Despite the term *dynamic*, the exploration is entirely passive, as the patient remains relaxed while the examiner manipulates his leg and applies the medial force.

### *Arthroscopic Evaluation*

**Figure 4:**
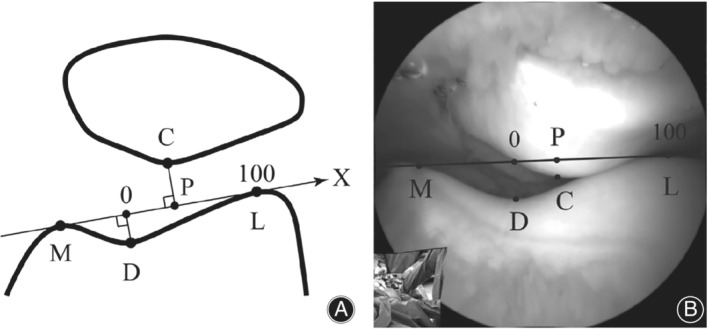
Arthroscopic assessment of lateral patellar translation by Suganuma^16^.

Before the routine implementation of high definition imaging devices such as the MRI, arthroscopy was considered the gold standard for intraarticular evaluation^14^. In terms of dynamic evaluation, arthroscopy allows the surgeon to directly visualize the relations in PFJ while it moves passively or actively; in the latter, the procedure should be performed under local anaesthetic to permit active colaboration^15^. It has also been studied that quadriceps activation by means of electrical stimulation significantly affects the relations in the PFJ observed during arthroscopy in PFI patients^16^ (Fig. 4).

Brossman proposed a 3‐tier malalignment classification according to arthroscopic findings, achieving good correlation with cine‐MRI findings^14^. This classification may be useful as a qualitative grading system during an arthroscopic evaluation, but the fact that it correlates well with the nowadays widely accessible MRI examination may raise a reasonable doubt about its current convenience as an isolated diagnostic gesture.

Recently, in 2019, an Australian group published the utility of arthroscopic evaluation in PFI assessment^33^. They estimated the knee flexion angle (KFA) at which the patella engages with the central portion of the trochlea and found a significantly higher angle in unstable joints: a recommendation to consider a tibial tubercle distal transfer is made when a KFA >40° is found.

### *Imaging Techniques*

#### *Computed Tomography (CT) Studies*

**Figure 5:**
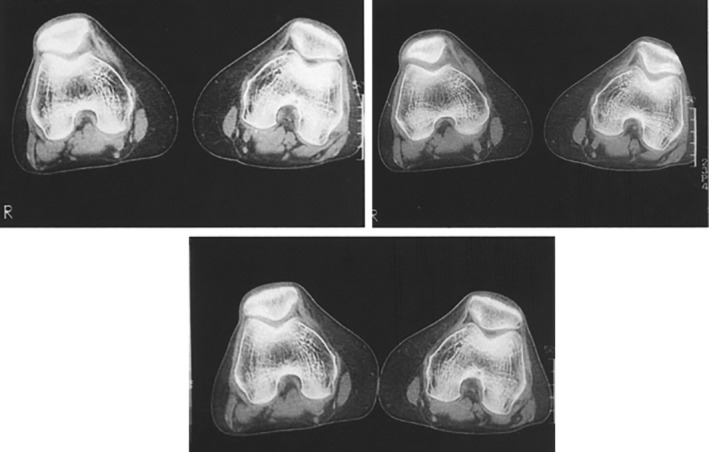
CT images obtained at different degrees of knee flexion and with/without quadriceps contraction^19^.

CT scans have been extensively used in the field of dynamic study of PFJ biomechanics. Early works in 1983–1986 accomplished dynamic sequences by obtaining static images with quadriceps contraction at several flexion angles, as motion capture techniques were not entirely available^17^, ^34^, ^35^. In 1988, the utility of electron‐beam CT for dynamic PFJ evaluation was reported; however, the high costs limited its availability^18^. In 1994, a study by Pinar, in which static images at different flexion angles were obtained, concluded: “*A regular pattern could not be observed. Further research is needed in this area”* and *“Kinematic CT scanning may reveal useful data on the pathogenesis of (…) PFI”* 19 (Fig. 5). Also in 1994, Guzzanti *et al*. observed that quadriceps activation during CT examination could displace the patella proximally up to 1 cm, potentially altering patellar height values, already suggesting that diagnostic and preoperative evaluations for PFI patients should not rely only on static imaging^20^.

Most succeeding efforts focused on dynamic kinematic CT scanning (DKCT). A first mention of a kinematic study of the PFJ by means of spiral CT dates back to 1997; the authors reported the feasibility of the technique and its clinical utility for PFI and anterior knee pain cases^36^. In recent years, we have witnessed a number of papers reporting on DKCT, both as a diagnostic tool and as an assessment method for surgical interventions for PFI. In 2014, Elias studied the biomechanical effects of surgical corrections in six PFI cases (medializing osteotomies and MPFL reconstruction) by means of kinematic CT^37^. Williams compared the results of dynamic CT scans from PFI knees to those of healthy contralaterals. The study was performed under no‐WB and described statistically significant differences in most parameters^38^. The same group also demonstrated how DKCT can differentiate specific patterns of patellar maltracking, establishing a grading system of 10 categories with high correlation with the severity of symptoms; only one of them was labelled as *normal tracking* 39. This variability was also highlighted in a specific review on patellar tracking, concluding that “*there may be no normal pattern*”^32^.

**Figure 6:**
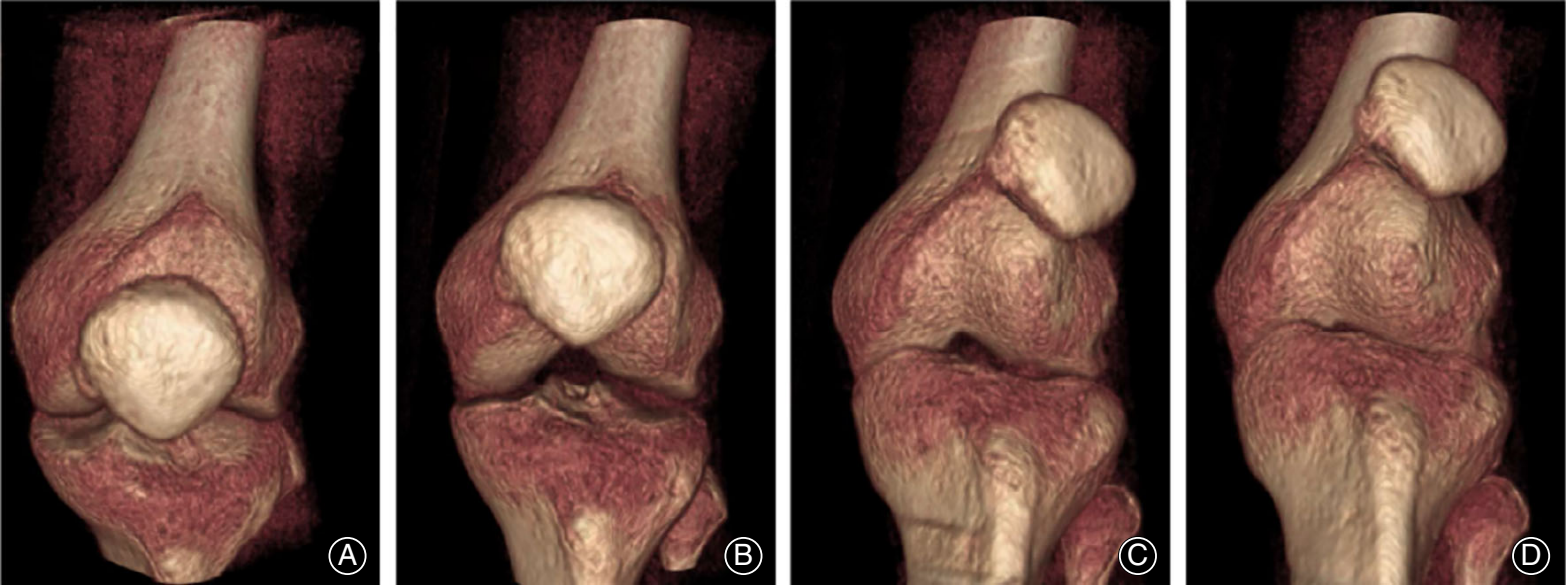
High definition 4DCT tracking pattern of a PFI knee by Forsberg *et al*.^21^.

Also, in 2016, Forsberg *et al*. reported another DKCT study assessing the results of isolated MPFL reconstructions, evaluating various methods to reference and compute anatomical landmarks^21^ (Fig. 6); another publication presented a similar design with the same purpose^22^.

#### *Dynamic MRI*

Several non‐static MRI protocols and devices have been employed in the analysis of PFJ (Table 3). The almost universal availability of this equipment, lack of radiation, and the progress in image definition and acquisition protocols have turned MRI into the preferred imaging option for knee clinicians.

**Table 3:** MRI modalities in PFJ assessment

Conventional MRI	Static image acquisition under isometric quadriceps contraction
Motion‐triggered cine MRI	Originally designed for cardiac studies, evaluates cyclic movement of the knee. Dependent on patient compliance, as multiples cycles have to be performed over several minutes at a certain pace.
Ultrafast MRI	Allows for image obtainment during slow motion of the knee. This avoids repetition of cyclic movements, decreasing study time and the need for specific patient collaboration and training.

A pathfinder paper, the first of a vast series, was published by Shellock in the late 1980s; he studied several aspects of PFJ biomechanics by means of “kinematic MRI,” obtaining cine‐sequences by combining static images acquired in increasing flexion angles^40^. The first publication of a motion‐trigger MRI study for PFJ dynamics appeared in 1993, reporting differences among normal and maltracking knees under active extension but not if passively extended^30^.

**Figure 7:**
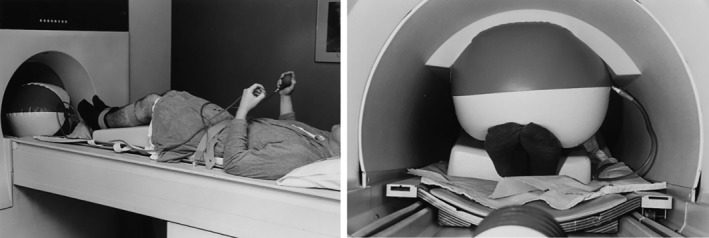
Inflatable device to allow for continuous isometric contraction during a range of knee flexion, by McNally^41^.

A first cine‐phase MRI report appeared in 1999, obtaining 3D motion studies from a healthy cohort under no‐WB; an error of up to 2° is noted^23^. The same year Witonski and Goraj published a clinical work employing dynamic MRI; in an anterior‐knee‐pain cohort they obtained conventional MRI sequences under isometric quadriceps contraction, in different degrees of knee flexion, and evaluated *sulcus angle, congruence angle and patellar tilt*, observing a significant difference to static images^10^. To our knowledge, the first study employing an ultrafast MRI protocol on PFI patients is the one from McNally *et al*. in 2000^41^; they found that a sulcus depth of less than 4 mm or with an angle flatter than 150° is highly specific (98%) for maltracking, providing clinically useful references (Fig. 7). Not later, O'Donnell *et al*. presented a paper *on MRI patellar tracking*, with an ultrafast protocol that reduced the examination time to 2 min^42^. They suggested no‐WB as a limitation in the study; at that time, some authors somehow managed to perform WB MRIs with the aid of custom‐made plastic supports, obtaining limited results but proving an increase in patellar cartilage contact area^43^. Evolutions were detailed in a 2009 work, when active sequences under WB were obtained while knees extended from 60 degrees of flexion to full extension^24^.

Several studies compared measurements obtained from static and dynamic MRI. Freedman and Sheehan evaluated patients with a history of maltracking, concluding than isolated static MRI would lead to a high rate of false negatives within his cohort^31^. Another study from Teng *et al*.^25^ observed the biomechanics of the PFJ under WB at different flexion angles. Notably, they observed the lateral trochlear inclination was the best predictor of patellar malalignment at all flexion angles, an outcome not observed during static MRI, stressing the relevance of dynamic evaluations. Recently, Carlson *et al*. studied the correlation between static obtained tibial tubercle–trochlear groove (TTTG) distance and cine‐MRI patellar tracking, concluding static TTTG is not a good predictor for lateral tracking^44^. This conclusion may alert surgeons accustomed to include it in their surgical planning^44^.

In addition, Burke *et al*. reported a continuous, real time radial gradient echo imaging for the first time in peripheral skeleton, obtaining high detail images and videos in moving knees from 0° to 30° of flexion, with a study time of 3–7 min^45^. They suggest a lateral subluxation beyond 3 mm could be considered as a PFI threshold, as no control subjects exceeded this value.

In 2016, by means of dynamic MRI assessment, Beckert *et al*. concluded that the modified lateral patellar edge measurement previously described by McDermott was a better target for surgical correction than the commonly used TTTG, which is generally obtained in a static CT examination^9^.

**Figure 8:**
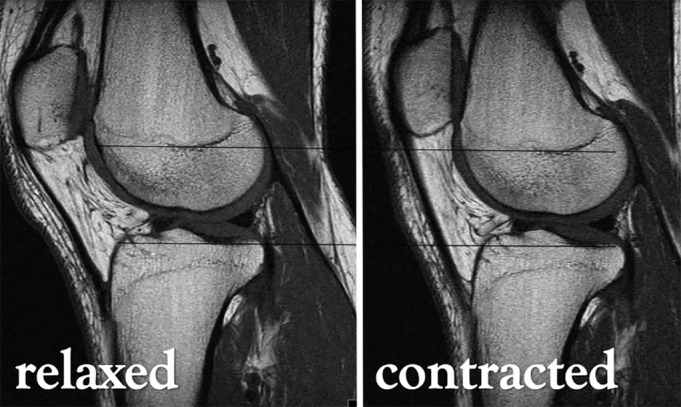
Proximal migration of the patella between conventional (left) and quadriceps contracted (right) sagittal MRI sequences, from Barroso *et al*.^26^.

Recently, the authors of this review published a novel technique to assess patellar height: the quads active ratio^26^. A dynamic MRI sequence was performed during volitional isometric quadriceps contraction, calculating the overlap among patellar and trochlear articular cartilages in a midsagittal slice: a quads active ratio of 0.12 showed good sensitivity for PFI^26^ (Fig. 8).

The feasibility of ultrafast dynamic MRI in paediatric population has also been tested, reporting compliant cooperation from a cohort aged 11–16 years; the evaluation was capable to identify differences between normal and unstable PFJs^46^. A protocol combining data from static high resolution MRI and low resolution active sequences of MRI was reported in another paediatric cohort, with a study time close to an hour^47^.

#### *Ultrasound (US)*

The development of high definition US devices and techniques has allowed for a more precise evaluation of musculoskeletal anatomy, and the PFJ has not been an exception.

**Figure 9:**
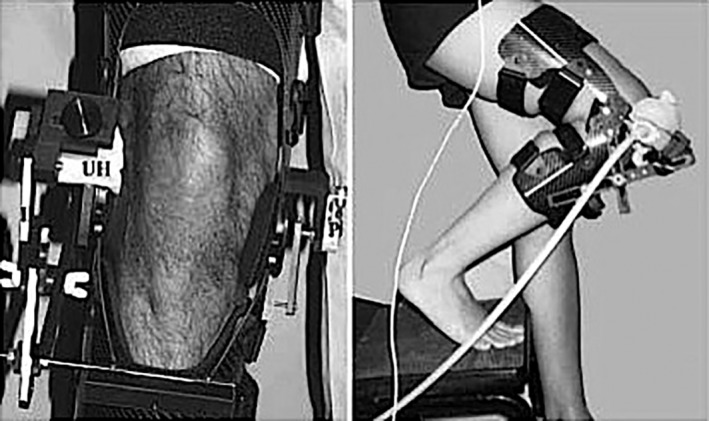
Shih custom‐made device attaching an ultrasound probe to a knee brace^27^.

Shih *et al.* modified a standard articulated knee brace with the addition of an US probe and a goniometer^27^ (Fig. 9). They evaluated PF tracking during several active knee movements (sitting, stepping, squatting…) and found significant differences in lateral tracking. The system was validated with MRI and proved good inter and intra‐rater reliability and complies with most of the ideal features listed by Muhle *et al*.^8^, but as the study only included healthy individuals, its clinical utility should be considered with caution.

Herrington's research group employed US to measure the distance between the lateral border of the patella and the edge of the lateral femoral condyle, while the quadriceps were contracted by means of electrical stimulation. Measurements were reported as reliable and reproducible, but PFI cases were excluded from the cohort^48^.

#### *Combined Imaging Techniques*

**Figure 10:**
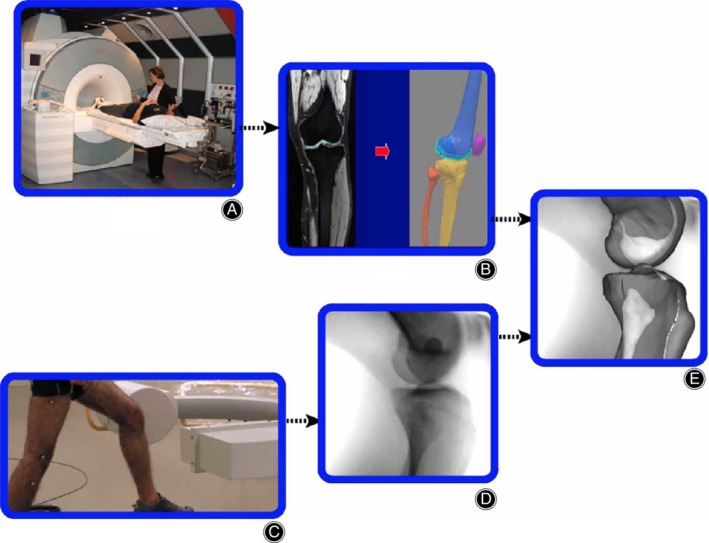
Multi‐source integrating algorithm by Fernandez *et al*.^49^.

A recurring approach in the study of the PFJ biomechanics has been the combination of data obtained by different imaging modalities. Most studies blend CT/MRI static data with the tracking patterns obtained with continuous fluoroscopy. Fernandez *et al*. pioneered in 2008 with a method fusing 3DMRI, dynamic 2D fluoroscopy, and video recording^49^ (Fig. 10). The paper described a pilot investigation in a healthy volunteer, with over 6 hours of processing time, but the accuracy is reported of less than 2 mm and 1° of error. Shortly later, Nha published a larger study applying a combination of static 3DMRI and 2D fluoroscopy during full range of movement WB squatting on 8 knees, validating the accuracy of the system with a cadaveric study^28^. This work produced a broad list of normal angles and measures, which probably have a better utility in the research field rather than in clinical practice.

More recently, an optimized protocol to join data from 3DMRI and 2D fluoroscopy was published, improving success rates and processing time with fewer errors^51^. The processing time ranged from 48–177 min, a significant reduction compared to previous works. In 2018, a new trial was conducted comparing the PFJ biomechanics of patients with anterior knee pain and volunteers, by a computerized combination of static CT images and dynamic fluoroscopy^52^.

### *Other Modalities*

**Figure 11:**
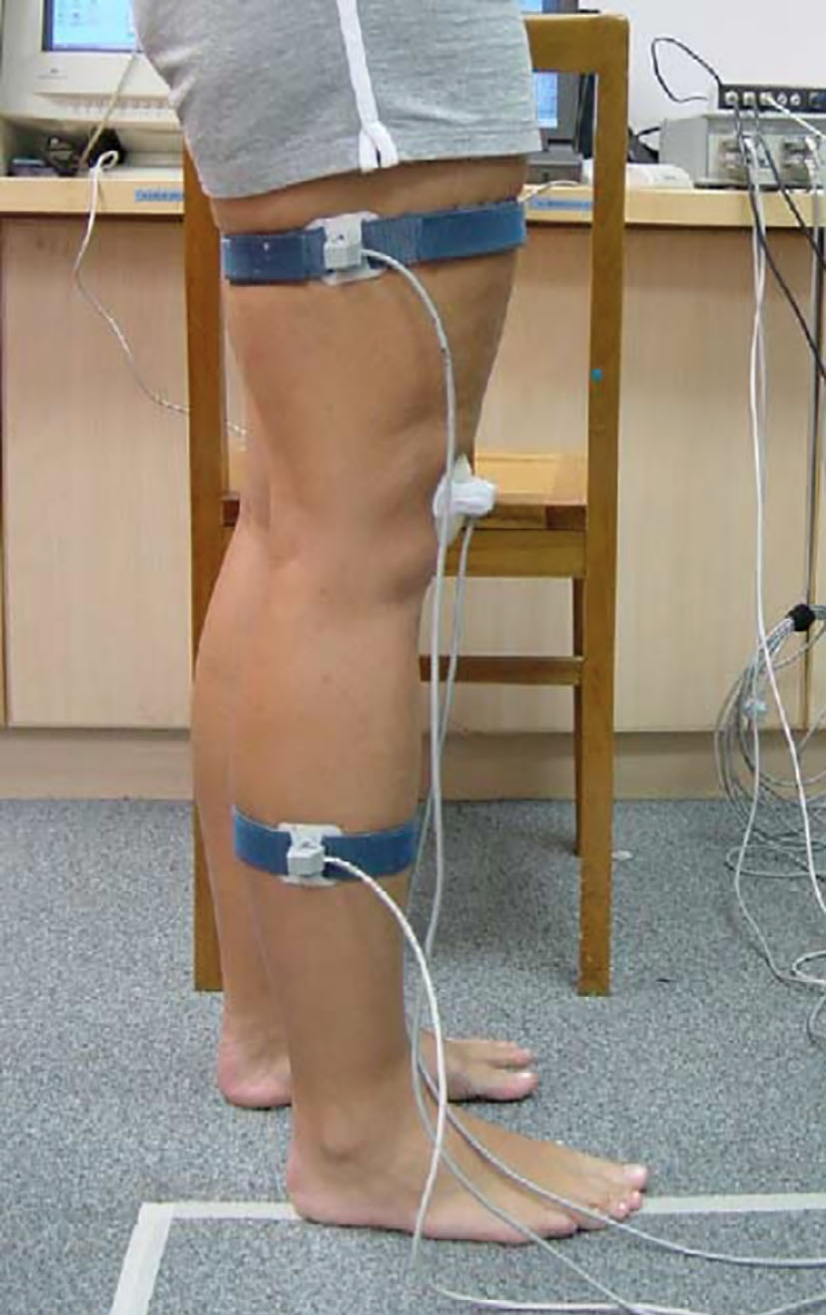
Non‐invasive sensor setting in Laprade and Lee's article^53^.

Additionally, distinct alternatives have been wielded in the dynamic study of the PFJ. Laprade and Lee conducted research on volunteers, assessing PFJ kinematics by means of a non‐invasively magnetic tracking system, attaching sensors to the skin; this device potentially allows for evaluation during any action^53^ (Fig. 11). Another interesting device was presented by Wilson *et al*.54; by means of a custom‐made thermoplastic patellar clamp attached to a goniometer, patellar motions were recorded by an optoelectronic capture system. This allowed obtaining data from real‐time squats: patellar extension, spin, tilt, and shift, with <1.2 and <1.1 mm error according to the cadaveric validation tests.

In 2016, Fujita *et al*. described an accessible method to qualitatively register PFJ tracking patterns by means of a conventional video camera and processing software^55^.

It is worth mentioning a paper by Liu *et al*. regarding the utility of diffusion tension imaging (DTI)^56^. This MRI‐based technique is mainly employed in neuroimaging, as it provides information about location, orientation, and anisotropy of neural tracts, but can also evaluate microscopic changes in muscular fibers. Comparing the data obtained from the vastus oblique medialis in a PFI cohort and in healthy controls, DTI was capable of detecting changes in muscular quality, even with no differences in muscle cross‐section volume. This could be considered as a screening test to detect early changes in PFI‐prone patients.

Recently, several research teams have employed wearable devices to study real time biomechanics of various anatomical segments, such as the lumbar spine^57^ or the knee^58^. There is great availability of these wireless devices, generally synchronized to mobile phones, and with accessible prices. They allow for long, unsupervised observation periods, as the subjects carry them during their usual activities within the community, which has proved useful to monitor adherence and performance in exercise and rehabilitation protocols^59^. These devices may become an interesting source of data for the dynamic evaluation of the PFJ in the near future, providing information during strenuous activities and sports practice.

### Conclusion

We believe future efforts should be pointed towards standardizing research protocols in order to obtain larger series of comparable data. Our impression is that the accuracy of many techniques cited in this paper, especially dynamic/kynematic MRI and DKCT, would warrant conclusive evidence to better understand the PFJ, but this should come from large series, including clinically affected PFI patients. Theoretically, dynamic assessment of the PFJ should be superior to the still established static methods. Many disparate publications point towards this direction, but the evidence is still insufficient to challenge current clinical practice. As seen in this review, most dynamic assessment modalities are not restricted to highly specialized centers and should be accessible and affordable in the utmost health settings. Stronger evidence on the clinical relevance of these investigations is warranted to advocate for their standardization in the study of clinically relevant PFI.
